# Engineering improved bio-jet fuel tolerance in *Escherichia coli* using a transgenic library from the hydrocarbon-degrader *Marinobacter aquaeolei*

**DOI:** 10.1186/s13068-015-0347-3

**Published:** 2015-10-07

**Authors:** Timothy A. Tomko, Mary J. Dunlop

**Affiliations:** School of Engineering, University of Vermont, 33 Colchester Ave, Burlington, VT 05405 USA

**Keywords:** Biofuel tolerance, *Marinobacter aquaeolei*, YceI, Pinene, Monoterpene, Genomic library, Transgenic

## Abstract

**Background:**

Recent metabolic engineering efforts have generated microorganisms that can produce biofuels, including bio-jet fuels, however these fuels are often toxic to cells, limiting production yields. There are natural examples of microorganisms that have evolved mechanisms for tolerating hydrocarbon-rich environments, such as those that thrive near natural oil seeps and in oil-polluted waters.

**Results:**

Using genomic DNA from the hydrocarbon-degrading microbe *Marinobacter aquaeolei*, we constructed a transgenic library that we expressed in *Escherichia coli*. We exposed cells to inhibitory levels of pinene, a monoterpene that can serve as a jet fuel precursor with chemical properties similar to existing tactical fuels. Using a sequential strategy with a fosmid library followed by a plasmid library, we were able to isolate a region of DNA from the *M. aquaeolei* genome that conferred pinene tolerance when expressed in *E. coli*. We determined that a single gene, *yceI*, was responsible for the tolerance improvements. Overexpression of this gene placed no additional burden on the host. We also tested tolerance to other monoterpenes and showed that *yceI* selectively improves tolerance.

**Conclusions:**

The genomes of hydrocarbon-tolerant microbes represent a rich resource for tolerance engineering. Using a transgenic library, we were able to identify a single gene that improves *E. coli*’s tolerance to the bio-jet fuel precursor pinene.

**Electronic supplementary material:**

The online version of this article (doi:10.1186/s13068-015-0347-3) contains supplementary material, which is available to authorized users.

## Background

Microorganisms are capable of producing advanced biofuels that can be used as ‘drop-in’ alternatives to conventional petroleum-based liquid fuels [1–3]. However, many of these fuels are toxic to cells, introducing an undesirable trade off between cell survival and biofuel production. There are many natural examples of microorganisms that survive in hydrocarbon-rich environments [4, 5]. We asked whether the genomes of these microbes could be a source of biofuel tolerance mechanisms when expressed heterologously in a biofuel production host. Microbes that tolerate hydrocarbons have been isolated near natural oil seepages and around oil spills. For example, a hydrocarbon-degrading microbe, *Marinobacter aquaeolei* VT8, was isolated at the head of an offshore oil well in Vietnam [6]. A previous study showed that this organism harbors two efflux pumps that can serve to improve biofuel tolerance [7]. That study also identified an efflux pump from *Alcanivorax borkumensis*, which is another hydrocarbon-degrader that thrives in oil-polluted waters. In addition to the ability to metabolize hydrocarbons, *A. borkumensis* possess multiple tolerance mechanisms, including the production of biosurfactants, efflux pumps, and niche-specific stress responses [8]. Studies of the microbial communities in the Gulf of Mexico after the Deepwater Horizon oil spill showed a high proportion of γ-Proteobacteria harboring hydrocarbon-degrading genes [9]. Given the abundance of naturally hydrocarbon-tolerant microorganisms, we hypothesized that the genomes of these organisms may serve as an untapped reservoir of tolerance genes.

In this study we focused specifically on improving tolerance to bio-jet fuels. Gasoline and diesel fuel can be supplemented with the use of battery power in terrestrial vehicles, however this is not a feasible option for aircraft, which have much more severe weight and size limitations. Aviation fuel must have a low enough freezing point that the fuel does not gel at low temperatures associated with typical flight altitudes. Also, the fuel must burn cleanly so as to not produce large amounts of soot that could potentially damage the turbine [10]. These considerations make bio-jet fuels a promising, but challenging, class of biofuels to produce. Monoterpenes (C_10_H_16_) such as pinene, limonene, terpinene, and terpinolene are composed of two isoprene units [10] and have been shown to be excellent candidates for replacements to commercial Jet-A/A-1 fuels [11–13]. Importantly, several of these bio-jet fuels have been produced by engineered microbial hosts. For example, in 2014, Sarria, et al. reported a pinene production pathway in *E. coli* [14]. Limonene production pathways have also been engineered in *E. coli* [7, 15]. Here, we focus initially on pinene as a representative bio-jet fuel precursor and later expand to other monoterpenes. Pinene dimers have similar chemical properties to the tactical jet-fuel JP-10 and can be produced synthetically in *E. coli* [12, 14].

A major challenge in the production of advanced biofuels is that toxicity limits the concentration of biofuel a cell can withstand. Vital physiological processes and membrane properties are often disrupted by the presence of these hydrocarbons; for reviews on mechanisms of biofuel toxicity and engineering approaches to mitigating toxicity see [16–19]. Previous tolerance engineering efforts have identified several ways that biofuel tolerance can be increased. For instance, export pumps, including efflux pumps and ABC transporters, can be used to improve biofuel tolerance and export [3, 7, 20, 21]. Additionally, several studies have shown that heat shock proteins can play an important role in improving solvent tolerance; recent examples include [22–24]. General stress response proteins can also mitigate biofuel toxicity and have appeared in screens for tolerance genes [18, 25].

Genomic library approaches have been successful at selecting for genes that improve tolerance. Typically, genomic DNA is either digested or sheared into fragment sizes appropriate for subcloning, inserted into the vector of choice, and transformed into the host. Cells containing members of the library are then subjected to a stressor and those that survive are screened using microarrays or sequencing. Most experiments related to biofuel tolerance that have used this approach use autologous libraries (for example, using genomic DNA from *E. coli* to screen in *E. coli*). Woodruff et al. used a multi-Scalar Analysis of Library Enrichments (SCALEs) approach to identify nine novel target genes to improve ethanol tolerance in *E. coli* [26]. Other studies involving the overexpression of endogenous genes have shown improvements in isobutanol and ethanol tolerance in *Saccharomyces cerevisiae* [27], and ethanol [28] and *n*-butanol [22] tolerance in *E. coli*. A small number of studies have used transgenic DNA libraries to improve tolerance in processes related to biofuel production. Zingaro et al. identified several tolerance mechanisms from *Lactobacillus plantarum,* that when expressed in *E. coli* show improved survival and growth under ethanol stress [29]. Ruegg et al. increased ionic liquid tolerance in an *E. coli* host by screening a fosmid library from *Enterobacter lignolyticus* for tolerance genes [30]. Genomic libraries represent an efficient way of screening many genes for desirable traits and transgenic libraries extend this approach to a potentially rich resource of tolerance genes.

There are many examples of studies where screening for genes that improve tolerance to exogenous addition of biofuel has proved successful in identifying tolerance genes that also improve biofuel yields. For example, in a recent study of isopentanol tolerance in *E. coli*, six out of eight of the genes that were found to improve tolerance to exogenous biofuel also increased production titers [31]. An efflux pump that was identified to improve tolerance to limonene also increased yield in a production strain [7]. A study performed in *Saccharomyces cerevisiae* showed that improved ethanol tolerance translates to a more efficient glucose to ethanol conversion rate [32]. However, there are counterexamples where improved tolerance does not lead to an increase in biofuel production, for example Atsumi et al. identified mutations that improved isobutanol-tolerance in *E. coli*. However their mutants did not yield higher titers of the biofuel product [33]. Due to the burden biofuel production pathways place on the cell, it is often more straightforward to screen for tolerance improvements with exogenous biofuel addition and later incorporate the tolerance strategy into a production strain. This approach has proved successful in many previous studies, and in this work we screen for pinene tolerance using exogenous addition of the biofuel.

Our overall goal was to identify novel biofuel tolerance genes from a hydrocarbon-tolerant microbe that could be used in *E. coli*. Using a transgenic library approach, we first used a fosmid library with *M. aquaeolei* VT8 genomic DNA inserts and isolated a single fosmid that improved pinene tolerance. We then made a plasmid sub-library from this fosmid DNA, which narrowed the field to two candidate genes. These genes were subcloned into new plasmids and tested for tolerance improvements. We identified a single gene, *yceI*, which provides improvements in pinene tolerance. Importantly, expression of *yceI* was not toxic under any of induction conditions we tested. The tolerance improvements also extended to another monoterpene, terpinolene, though not limonene or terpinene. In this study, we describe an approach for bioprospecting for new tolerance genes from microbes that survive in hydrocarbon-rich environments. As a proof of principle, we show that we are able to identify a single gene that can improve tolerance to the bio-jet fuel pinene without introducing additional toxic effects.

## Results and discussion

**Fig. 1:**
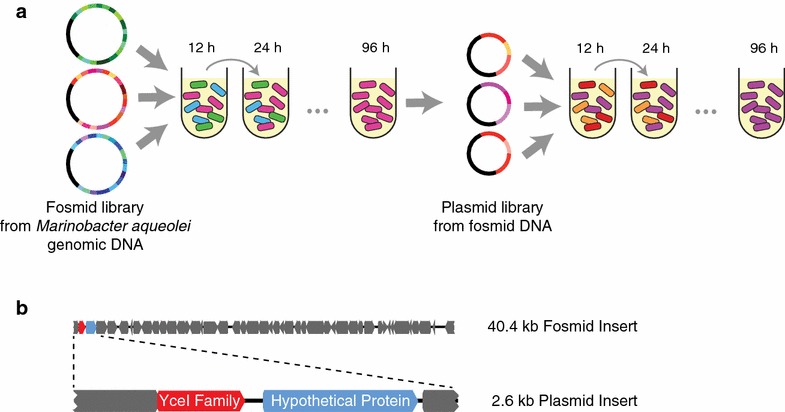
Genomic library approach and sequences isolated in selection experiments. **a** Illustration of the selection experiment used to isolate tolerance genes from *M. aquaeolei*. *E. coli* containing fosmids with *M. aquaeolei* inserts were grown in the presence of 0.05 % (v/v) pinene. Every 12 h, the cultures were diluted into fresh media with pinene. The fosmid that survived the initial selection experiment was then used as a template for a plasmid library and the selection procedure was repeated. **b** The isolated fosmid and plasmid inserts

To screen and identify genes that confer biofuel tolerance to *E. coli*, we created a transgenic library from *M. aquaeolei* VT8 genomic DNA (Methods). To provide good coverage and potentially include complex biofuel tolerance mechanisms that involve multiple genes we used relatively large (~40 kb) inserts to construct a fosmid library, which we screened in *E. coli*. Based on colony counts, we estimate that our library covers 100 % of the *M. aquaeolei* genome with 99.9 % certainty [34] (“Methods”). The *M. aquaeolei* fosmid library was screened in 0.05 % (v/v) pinene over the course of 96 h, using serial dilutions into fresh media with pinene every 12 h (Fig. 1a). We selected this pinene concentration because it severely inhibited growth of the negative control, while cultures with the library survived (see Additional file 1: Fig. S1). In principle, cells that contain the most beneficial transgenic DNA in the population stressed with pinene should outcompete other cells that do not have advantageous genes. We conducted parallel control experiments where we grew the library in the absence of pinene; we also included a fosmid negative control, where we grew cells without the library in the presence of pinene. The latter controls for *E. coli* genomic mutations that may arise due to pinene stress. We verified that cultures grown from a single colony isolated at the final time point were able to grow better in pinene than the samples with the control fosmid (see Additional file 2: Fig. S2).

At the 0, 24, 48, and 96-h time points we plated cells to isolate single colonies, extracted fosmids, and sequenced to check for library convergence. As expected, at 0 h all three samples that were sequenced were unique since no stress had been applied to the library yet (see Additional file 3: Table S1). Again, after 24 h, all sequenced samples were unique with no overlap between the inserts. By 48 h the three sequenced samples were identical and matched those that were subsequently extracted at the 96-h time point. We note that one of the three fosmids sequenced from the 24-h time point overlapped the fosmid sequence from 48 and 96 h, but the start and end points of the sequence differed. We refer to the converged fosmid here as pCC1FOS-96. The insert contained in pCC1FOS-96 had 43 complete genes from *M. aquaeolei*. The next step was to determine which gene or genes were conferring pinene tolerance to *E. coli.*

To efficiently screen for beneficial genes, we created a sub-library using the fosmid pCC1FOS-96 as the starting template. pCC1FOS-96 DNA was partially digested using the restriction enzyme Sau3A1. Cut sites for Sau3A1 (GATC) appear frequently in the sequence, so we used a partial digest, controlling the length of the reaction and the units of enzyme added (“Methods”). We extracted DNA in the 4–12 kb range, selecting this size to allow for complete genes or the possibility of multiple genes without creating inserts that would be prohibitive for cloning or those that return so many genes that it would be difficult to determine contributions. We cloned the inserts into the medium copy vector pBbA5k [35], using *E. coli* MG1655 for all subsequent experiments. In order to screen for genes that conferred tolerance, we repeated the selection procedure, stressing cells with 0.05 % pinene over the course of 96 h, as we did with the fosmid library (Fig. 1a). We conducted the same control experiments as in the fosmid library, replacing the negative control fosmid with pBbA5k-rfp, which expresses red fluorescent protein and does not confer pinene tolerance. Upon completion of the selection experiment, all colonies sampled contained identical plasmids, which we refer to as pBbA5k-96. We extracted and retransformed this plasmid into fresh *E. coli* MG1655 cells to control for the possibility that improvements in tolerance were due to genomic mutations, however the freshly transformed cells retained improved tolerance. This indicated that the presence of the pBbA5k-96 plasmid was responsible for conferring tolerance.

**Fig. 2:**
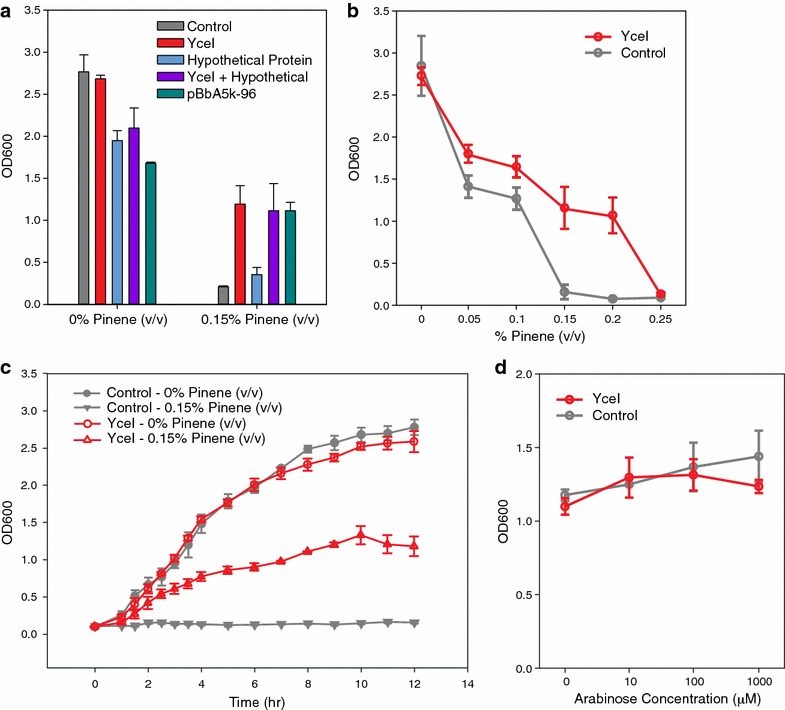
*yceI* is the gene responsible for pinene tolerance and its overexpression is not toxic.** a** Testing the tolerance of cells containing plasmids with the individual genes identified in the pinene tolerance selection. The control is pBbA8k-rfp; other plasmids use the pBbA8k vector and contain either *yceI*, the gene encoding the hypothetical protein, or both. pBbA5k-96 contains both genes as well as the truncated genes at the start and end of the plasmid insert (see Additional file 3: Table S1). Cells were induced using 100 µM arabinose. **b** Final OD values under conditions with increasing pinene levels. **c** Growth curves of cells expressing *yceI* (pBbA5k-yceI) compared to the control (pBbA5k-rfp) in 0 % and 0.15 % pinene. Cells were induced using 100 µM arabinose. **d** Measuring the toxicity of *yceI* expression compared to the control by inducing expression with arabinose. In all plots, error bars represent one standard deviation from the mean

The plasmid pBbA5k-96 contains two complete genes from *M. aquaeolei* with additional truncated genes on each side (Fig. 1b). The complete genes encode for a YceI-family protein, Maqu_1680 [GenBank:YP_958951.1], and a hypothetical protein, Maqu_1681 [GenBank:YP_958952.1]. To determine which gene or genes were responsible for providing increased pinene tolerance, we subcloned the two complete genes individually and together into pBbA8k medium copy vectors [35]. The plasmid contains an arabinose-inducible P_BAD_ promoter, allowing us to control the level of gene expression. Using 100 µM arabinose we tested the tolerance of cells containing plasmids with the individual and combined genes (Fig. 2a). Under no pinene stress, cells with YceI grew comparably to the negative control, while all cells expressing the hypothetical protein (alone, in combination with YceI, and on the original pBbA5k-96) experienced a reduction in growth. When exposed to 0.15 % pinene, only cells with the YceI protein showed improvements in growth relative to the negative control. Statistical analysis showed a significant benefit to having YceI in the samples when pinene is both present and absent (*p* value <0.001, ANOVA with Dunnett’s test). There was no statistical difference between samples with and without the hypothetical protein in the presence of pinene.

YceI-family proteins are diverse and remain largely uncharacterized. The YceI-like protein TT1927B from *Thermus thermophiles* HB8 functions as an isoprenoid transport or storage protein and may serve as a part of an unknown isoprenoid metabolic pathway [36]. The crystal structure for the protein was solved with the protein complexed with its ligand, a C_40_ isoprenoid. HP1286 from *Helicobacter pylori* is another YceI-like protein and is overexpressed in acid stress [37]. It binds to amphiphilic compounds containing approximately 22 carbon atoms. HP1286 is secreted by the cell, potentially to sequester and supply fatty acids from the environment for metabolism or to detoxify and protect the cell from the antimicrobial properties of fatty acids. *E. coli* harbors a periplasmic YceI-like protein that responds to basic, high pH conditions, but its function has not otherwise been characterized [38]. YceI from *M. aquaeolei* shares 36 % identity with TT1927b, a 31 % identity with HP1286, and a 35 % identity with the YceI-like protein from *E. coli*. Previously studied proteins in the YceI family have a multitude of functions, ranging from a potential role in the isoprenoid metabolic pathway to involvement in acid or base-induced stress.

We next tested different concentrations of pinene to determine the highest level cells expressing *yceI* could withstand (Fig. 2b). For all tested pinene levels up to 0.25 %, cells expressing *yceI* grew better than the control. Growth curves with 0.15 % pinene highlight differences in specific growth rates for strains with and without *yceI* (Fig. 2c). We also tested whether overexpression of *yceI* was toxic to cells. Other biofuel tolerance mechanisms, such as efflux pumps are known to provide a benefit under stress, but are toxic if overexpressed, leading to a trade off in expression levels for optimal cell survival [39]. We tested varying levels of inducer ranging from 0 to 1000 µM arabinose and observed no growth impact compared to the control at any concentration of inducer (Fig. 2d). For all concentrations of arabinose, growth of cells expressing the *yceI* gene were statistically equivalent to the negative control (one-way ANOVA test).

**Fig. 3:**
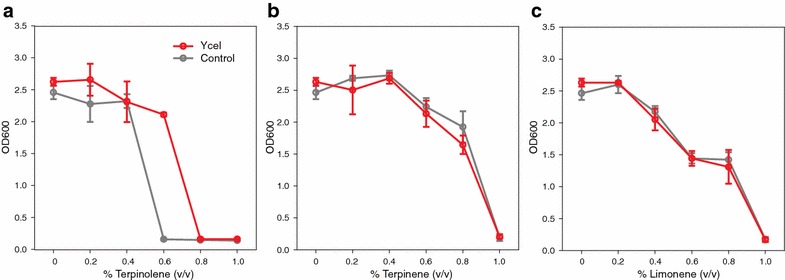
Testing the tolerance effects of *yceI* in the bio-jet fuel precursors **a** terpinolene, **b** terpinene, and **c** limonene. *Error bars* correspond to one standard deviation from the mean, measured 12 h after the addition of biofuel

Due to the similar chemical structure of pinene to the bio-jet fuel precursors terpinolene, terpinene, and limonene [11], we tested tolerance using these chemicals as well. Cells expressing *yceI* were stressed in increasing concentrations of the three monoterpene fuels. Our results indicate that *yceI* was beneficial in improving growth when cells were exposed to terpinolene (Fig. 3a), however for the two other fuel precursors tested, limonene and terpinene, we saw no significant improvement over the control (Figs. 3b, c).

## Conclusions

In this study, we used a library approach to screen the genome of the hydrocarbon-degrading microbe *M. aquaeolei* for genes that impart tolerance to potential bio-jet fuel precursors. We showed that expression of the gene *yceI* from *M. aquaeolei* successfully increases pinene and terpinolene tolerance when expressed in *E. coli* MG1655. Based on previous accounts in the literature, *yceI* may act by sequestering or transporting chemicals within the periplasmic space, thus alleviating some degree of biofuel toxicity. However, this protein family is poorly characterized and in the future it will be interesting to further explore the function of *M. aquaeolei yceI* in *E. coli*. We see no evidence of toxicity associated with the overexpression of *yceI* in our experiments, suggesting that this may be a novel, low-burden mechanism for improving pinene tolerance.

In the future it will be interesting to test *yceI* in a pinene production strain to determine the potential titer increase that could be achieved. Also, combining *yceI* with other known tolerance mechanisms has the potential to synergistically increase cell survival at higher biofuel concentrations. This has been shown in the past to be successful in boosting tolerance to higher levels than could achieved by one mechanism alone [39].

This method of library construction and competition could also be used to discover additional tolerance genes. Other hydrocarbon resistant microbes such as *A. borkumensis* or the strains isolated during the Deepwater Horizon disaster, would make excellent sources of genetic material for library construction. Alternatively, instead of selecting individual microbes for library screening, a metagenomic approach could be applied. Gathering samples of microbial populations from the environment has the potential to provide a diverse sample of genomic DNA.

Given the importance of liquid fuels in our society, it is critical to ensure that we have reliable and economical sources of these fuels for the future. Identifying better ways to produce advanced biofuels will help to lower the cost of their production and make biofuels more competitive with petroleum-derived fuels in the future.

## Methods

### Fosmid library construction

*M. aquaeolei* VT8 genomic DNA was obtained from ATCC (**#**700491D-5) and was used for the construction of the fosmid library. The degree of genomic DNA fragmentation was determined via gel electrophoresis on a 0.7 % agarose gel run for 20 h at 30 volts. The gel was then stained and imaged. The genomic DNA had an average length of ~40 kb, which we used directly as the insert in the library construction process. The fosmid library was created using the CopyControl Fosmid Library Production Kit with the pCC1FOS vector (Epicentre). A control fosmid was also created using the ~42 kb fragment of human X-chromosome DNA provided as a control in the kit. *E. coli* EPI300-T1 cells were mixed with the prepared phage particles at different concentrations to determine an appropriate titer, and we selected a 1:10 dilution. The *M. aquaeolei* library and control fosmid were plated on Luria–Bertani (LB) agar plates containing 12.5 µg/ml chloramphenicol, yielding approximately 1000 colonies per plate after overnight growth at 37 °C. Library coverage was determined using the Clark-Carbon equation, based on the assumption that recombinant clones follow a Poisson distribution across the genome [34]. Based on these calculations, there is 99.9 % certainty that the library has full coverage. The colonies containing fosmids with *M. aquaeolei* DNA were scraped off the plate using a razor blade and 50 µl aliquots of the library were stored in a 20 % glycerol solution at −80 °C for later use. An individual colony from the control fosmid plate was used to create a glycerol stock.

### Selection procedure: fosmid library

LB supplemented with 12.5 µg/ml of chloramphenicol was used to prepare two cultures containing *E. coli* EPI300-T1 cells harboring the *M. aquaeolei* fosmid library. A third culture containing the human DNA control fosmid in *E. coli* EPI300-T1 was prepared in a similar manner. One of the *M. aquaeolei* library cultures and the control were stressed with 0.05 % pinene (v/v), while the other *M. aquaeolei* library culture received no exposure to pinene. Each of the cultures was diluted 1:100 into LB plus chloramphenicol and pinene, where applicable, every 12 h over the course of a 96 h period. Every 24 h, samples from each culture were plated on LB agar plates with chloramphenicol. Three colonies from each of the 0, 24, 48, and 96-h time points were selected and their fosmids were extracted as follows: Cells were grown in 5 ml of LB medium plus chloramphenicol and 10 µl of 500X CopyControl Fosmid Autoinduction solution from the Epicentre Kit. The culture was grown for 16 h and the fosmid DNA was then extracted using a Qiagen QIAprep Spin Miniprep Kit. The fosmid samples were sequenced using the pCC1 forward (5′-GGATGTGCTGCAAGGCGATTAAGTTGG-3′) and reverse (5′-CTCGTATGTTGTGTGGAATTGTGAGC-3′) primers indicated in the Epicentre Kit protocol. The resulting sequences were aligned with the *M. aquaeolei* genome (see Additional file 3: Table S1). The converged fosmid from the 96-h time point, which we refer to as pCC1FOS-96, was saved for later use.

### Plasmid library construction

The fosmid from the converged 96-h time point (pCC1FOS-96) was used as the starting material for plasmid library construction. 500 µg of the fosmid DNA was partially digested at 37 °C using varying concentrations (0.13U, 0.25U, 0.38U, and 0.50U) of the enzyme Sau3A1 (New England Biolabs). By reducing the digestion time to five minutes, we achieved a DNA insert length centered around 4–12 kb. The digested fosmid DNA from each of the four reactions was then gel extracted from the 4–12 kb range using the Qiagen QIAquick Gel Extraction Kit, as in [40]. We used pBbA5k [35] as the plasmid vector, which has a medium copy p15A origin of replication, lacUV5 promoter upstream of the cloning site, and a kanamycin resistance gene. To prepare the vector, the pBbA5k-rfp plasmid was double digested using BamHI and BglII and gel extracted. The pBbA5k vector and prepared library inserts were ligated using T4 DNA ligase (Fermentas) at a 3:1 insert to vector ratio. The ligated mixture was transformed into *E. coli* MG1655 and plated onto LB agar plates containing 50 µg/ml kanamycin. Plates were incubated overnight at 37 °C and the resulting ~500 colonies were scraped off of the plates using a razor blade and stored at −80 °C in a 20 % glycerol solution, as described above.

### Selection procedure: plasmid library

The selection experiment run using the plasmid library was the same as the fosmid selection described above, with the following changes: pBbA5k-rfp in *E. coli* MG1655 was used as the control, 50 µg/ml of kanamycin was used in place of chloramphenicol, and sequencing was performed on three samples from the pinene-treated plasmid library plate at 96 h using the forward (5′-GGAATTGTGAGCGGATAACAATTTC-3′) and reverse (5′-CGTTTTATTTGATGCCTGGAGATCC-3′) primers for the pBbA5k vector, as given in [35]. The converged plasmid after 96 h was saved for later use and named pBbA5k-96.

### Subcloning genes from the converged plasmid

Two *M. aquaeolei* genes encoding for a YceI family protein [GenBank:YP_958951.1] and a hypothetical protein [GenBank:YP_958952.1] were subcloned using the pBbA8k BioBrick vector [35], which has a p15A origin of replication, P_BAD_ promoter, and kanamycin resistance cassette. The DNA fragments were cloned using the Gibson Assembly Protocol [41]. Inserts were prepared using PCR and the vector was prepared by digesting pBbA8k-rfp with BamHI and BglII and gel extracting. Individual colonies of each construct were isolated and cloning success was verified via sequencing using the forward (5′-CTACTGTTTCTCCATACCCGTTTTTTTGG-3′) and reverse (5′-CGTTTTATTTGATGCCTGGAGATCC-3′) primers for the pBbA8k vector [35].

### Tolerance testing procedure

Overnight cultures were grown for 16 h in LB containing antibiotics and varying levels of arabinose (as required). Cultures for tolerance testing were prepared by first preparing a pre-culture, where we inoculated 5 ml of LB containing antibiotics and arabinose (as required) with 50 µL of the overnight culture. Cultures were grown until they reached an OD600 reading of 0.2, at which point varying levels of biofuel were added to each. The chemicals used for tolerance testing were obtained from Sigma Aldrich (α-pinene P45680, γ-terpinene 86478, limonene 183164, and terpinolene W304603). Growth measurements were taken after 12 h under biofuel stress. For the growth curve experiments, measurements were taken every 30 min for the first 4 h; after this point OD600 readings were taken every hour. All experiments were performed in triplicate.

## Additional files

10.1186/s13068-015-0347-3 Initial testing of *E. coli* EPI300-TI cells containing the control fosmid and the *M. aquaeolei* fosmid library. 0.05 % pinene was selected for subsequent experiments.
10.1186/s13068-015-0347-3 Pinene tolerance of *E. coli* EPI300-TI cells containing the converged fosmid (pCC1FOS-96) in 0.2 % pinene measured after 16 h of growth.
10.1186/s13068-015-0347-3 Fosmid library convergence.

## Abbreviations

ABC transporter: ATP-binding cassette transporter
OD: optical density
PCR: polymerase chain reaction

## References

[1] Fortman JL, Chhabra S, Mukhopadhyay A, Chou H, Lee TS, Steen E (2008). Biofuel alternatives to ethanol: pumping the microbial well. Trends Biotechnol.

[2] Lee SK, Chou H, Ham TS, Lee TS, Keasling JD (2008). Metabolic engineering of microorganisms for biofuels production: from bugs to synthetic biology to fuels. Curr Opin Biotechnol.

[3] Fischer CR, Klein-Marcuschamer D, Stephanopoulos G (2008). Selection and optimization of microbial hosts for biofuels production. Metab Eng.

[4] Camilli R, Reddy CM, Yoerger DR, Van Mooy BAS, Jakuba MV, Kinsey JC (2010). Tracking hydrocarbon plume transport and biodegradation at Deepwater Horizon. Science.

[5] Rojas A, Duque E, Mosqueda G, Golden G, Hurtado A, Ramos JL (2001). Three efflux pumps are required to provide efficient tolerance to toluene in *Pseudomonas putida* DOT-T1E. J Bacteriol.

[6] Huu NB, Denner EB, Ha DT, Wanner G, Stan-Lotter H (1999). *Marinobacter aquaeolei* sp. nov., a halophilic bacterium isolated from a Vietnamese oil-producing well. Int J Syst Bacteriol.

[7] Dunlop MJ, Dossani ZY, Szmidt HL, Chu HC, Lee TS, Keasling JD (2011). Engineering microbial biofuel tolerance and export using efflux pumps. Mol Syst Biol.

[8] Schneiker S, dos Santos VAPM, Bartels D, Bekel T, Brecht M, Buhrmester J (2006). Genome sequence of the ubiquitous hydrocarbon-degrading marine bacterium *Alcanivorax borkumensis*. Nat Biotechnol.

[9] Hazen TC, Dubinsky EA, DeSantis TZ, Andersen GL, Piceno YM, Singh N (2010). Deep-sea oil plume enriches indigenous oil-degrading bacteria. Sci NY..

[10] Schobert HH. The chemistry of hydrocarbon fuels. Butterworth-Heinemann; 2013.

[11] Brennan TCR, Turner CD, Krömer JO, Nielsen LK (2012). Alleviating monoterpene toxicity using a two-phase extractive fermentation for the bioproduction of jet fuel mixtures in *Saccharomyces cerevisiae*. Biotechnol Bioeng.

[12] Harvey BG, Wright ME, Quintana RL (2010). High-density renewable fuels based on the selective dimerization of pinenes. Energy Fuels.

[13] Chuck CJ, Donnelly J (2014). The compatibility of potential bioderived fuels with Jet A-1 aviation kerosene. Appl Energy.

[14] Sarria S, Wong B, García Martín H, Keasling JD, Peralta-Yahya P (2014). Microbial synthesis of pinene.. ACS Synth Biol.

[15] Alonso-Gutierrez J, Chan R, Batth TS, Adams PD, Keasling JD, Petzold CJ (2013). Metabolic engineering of *Escherichia coli* for limonene and perillyl alcohol production. Metab Eng.

[16] Dunlop MJ (2011). Engineering microbes for tolerance to next-generation biofuels. Biotechnol Biofuels.

[17] Ramos JL, Duque E, Gallegos M-T, Godoy P, Ramos-Gonzalez MI, Rojas A (2002). Mechanisms of solvent tolerance in gram-negative bacteria. Annu Rev Microbiol.

[18] Nicolaou SA, Gaida SM, Papoutsakis ET (2010). A comparative view of metabolite and substrate stress and tolerance in microbial bioprocessing: from biofuels and chemicals, to biocatalysis and bioremediation. Metab Eng.

[19] Peabody GL, Winkler J, Kao KC (2014). Tools for developing tolerance to toxic chemicals in microbial systems and perspectives on moving the field forward and into the industrial setting. Curr Opin Chem Eng.

[20] Doshi R, Nguyen T, Chang G (2013). Transporter-mediated biofuel secretion. Proc Natl Acad Sci USA.

[21] Foo JL, Leong SSJ (2013). Directed evolution of an *E. coli* inner membrane transporter for improved efflux of biofuel molecules. Biotechnol Biofuels.

[22] Reyes LH, Almario MP, Kao KC (2011). Genomic library screens for genes involved in n-butanol tolerance in *Escherichia coli*. PLoS One.

[23] Fiocco D, Capozzi V, Goffin P, Hols P, Spano G (2007). Improved adaptation to heat, cold, and solvent tolerance in *Lactobacillus plantarum*. Appl Microbiol Biotechnol.

[24] Alsaker KV, Paredes C, Papoutsakis ET (2010). Metabolite stress and tolerance in the production of biofuels and chemicals: Gene-expression-based systems analysis of butanol, butyrate, and acetate stresses in the anaerobe *Clostridium acetobutylicum*. Biotechnol Bioeng.

[25] Weber H, Polen T, Heuveling J, Wendisch VF, Hengge R (2005). Genome-wide analysis of the general stress response network in *Escherichia coli*: S-Dependent genes, promoters, and sigma factor selectivity. J Bacteriol.

[26] Woodruff LBA, Pandhal J, Ow SY, Karimpour-Fard A, Weiss SJ, Wright PC (2013). Genome-scale identification and characterization of ethanol tolerance genes in Escherichia coli. Metab Eng.

[27] Hong M-E, Lee K-S, Yu BJ, Sung Y-J, Park SM, Koo HM (2010). Identification of gene targets eliciting improved alcohol tolerance in *Saccharomyces cerevisiae* through inverse metabolic engineering. J Biotechnol.

[28] Nicolaou SA, Gaida SM, Papoutsakis ET (2012). Exploring the combinatorial genomic space in Escherichia coli for ethanol tolerance. Biotechnol J.

[29] Zingaro KA, Nicolaou SA, Yuan Y, Papoutsakis ET (2014). Exploring the heterologous genomic space for building, stepwise, complex, multicomponent tolerance to toxic chemicals. ACS Synth Biol..

[30] Ruegg TL, Kim E-M, Simmons BA, Keasling JD, Singer SW, Lee TS (2014). An auto-inducible mechanism for ionic liquid resistance in microbial biofuel production. Nat Commun..

[31] Foo JL, Jensen HM, Dahl RH, George K, Keasling JD, Lee TS (2014). Improving microbial biogasoline production in Escherichia coli using tolerance engineering. MBio..

[32] Alper H, Moxley J, Nevoigt E, Fink GR, Stephanopoulos G (2006). Engineering yeast transcription machinery for improved ethanol tolerance and production. Science.

[33] Atsumi S, Wu T-Y, Machado IMP, Huang W-C, Chen P-Y, Pellegrini M (2010). Evolution, genomic analysis, and reconstruction of isobutanol tolerance in Escherichia coli. Mol Syst Biol.

[34] Clarke L, Carbon J (1976). A colony bank containing synthetic Col El hybrid plasmids representative of the entire *E. coli* genome. Cell..

[35] Lee TS, Krupa RA, Zhang F, Hajimorad M, Holtz WJ, Prasad N (2011). BglBrick vectors and datasheets: a synthetic biology platform for gene expression. J Biol Eng.

[36] Handa N, Terada T, Doi-Katayama Y, Hirota H, Tame JRH, Park S-Y (2005). Crystal structure of a novel polyisoprenoid-binding protein from *Thermus thermophilus* HB8. Protein Sci.

[37] Sisinni L, Cendron L, Favaro G, Zanotti G (2010). Helicobacter pylori acidic stress response factor HP1286 is a YceI homolog with new binding specificity. FEBS J.

[38] Stancik LM, Stancik DM, Schmidt B (2002). pH-dependent expression of periplasmic proteins and amino acid catabolism in *Escherichia coli*. J Bacteriol.

[39] Turner WJ, Dunlop MJ (2014). Trade-offs in improving biofuel tolerance using combinations of efflux pumps. ACS Synth Biol.

[40] Wargo MJ, Szwergold BS, Hogan DA (2008). Identification of two gene clusters and a transcriptional regulator required for *Pseudomonas aeruginosa* glycine betaine catabolism. J Bacteriol.

[41] Gibson DG, Young L, Chuang R-Y, Venter JC, Hutchison CA, Smith HO (2009). Enzymatic assembly of DNA molecules up to several hundred kilobases. Nat Methods.

