# Supplementary figures and images for: Engineering improved bio-jet fuel tolerance in Escherichia coli using a transgenic library from the hydrocarbon-degrader Marinobacter aquaeolei
Source: Biotechnol Biofuels. 2015 Oct 7;8:165. doi: 10.1186/s13068-015-0347-3 (PMC4596283; doi:10.1186/s13068-015-0347-3)

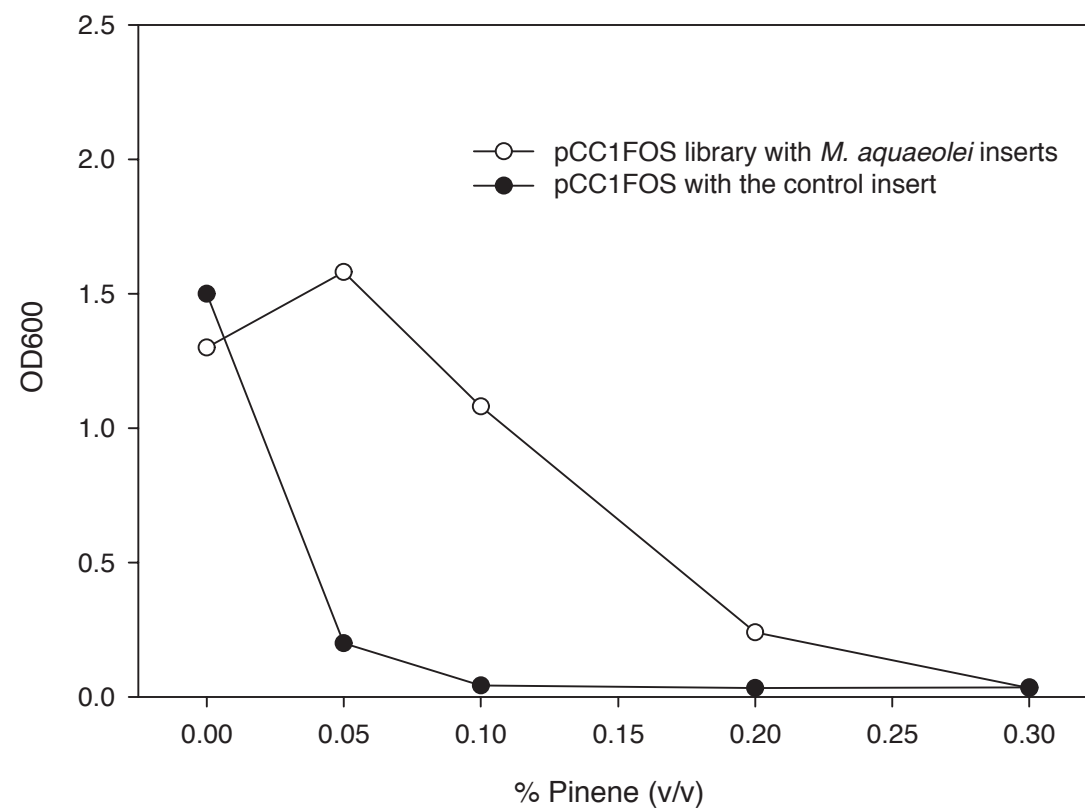

**Figure S1**

Supplement: Supplementary file 1 — 10.1186/s13068-015-0347-3 Initial testing of E. coli EPI300-TI cells containing the control fosmid and the M. aquaeolei fosmid library. 0.05 % pinene was selected for subsequent experiments. [file 13068_2015_347_MOESM1_ESM.pdf]

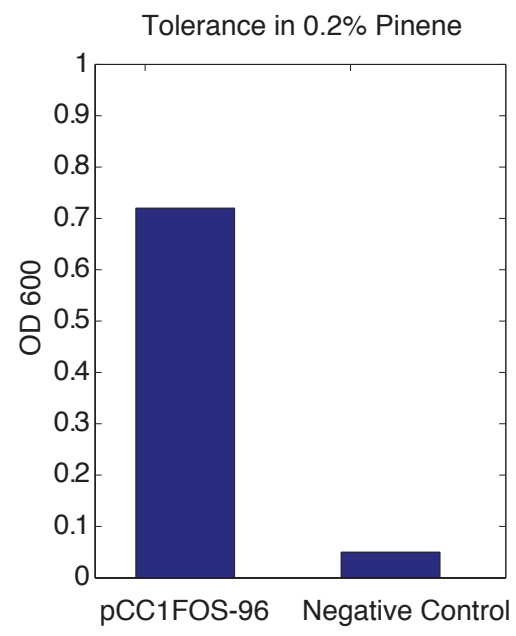

**Figure S2**

Supplement: Supplementary file 2 — 10.1186/s13068-015-0347-3 Pinene tolerance of E. coli EPI300-TI cells containing the converged fosmid (pCC1FOS-96) in 0.2 % pinene measured after 16 h of growth. [file 13068_2015_347_MOESM2_ESM.pdf]
