# Supplementary material for: Engineering improved bio-jet fuel tolerance in Escherichia coli using a transgenic library from the hydrocarbon-degrader Marinobacter aquaeolei
Source: Biotechnol Biofuels. 2015 Oct 7;8:165. doi: 10.1186/s13068-015-0347-3 (PMC4596283; doi:10.1186/s13068-015-0347-3)
Supplement: Supplementary file 3 — 10.1186/s13068-015-0347-3 Fosmid library convergence. [file 13068_2015_347_MOESM3_ESM.docx]

**Table S1. Fosmid library convergence**

Sequencing results from single colonies at various time points in the selection experiments. Fosmids and plasmids were sequenced using primers listed in Methods and the start and stop position on the *Marinobacter aquaeolei* genome were determined from these results. The numbers in the table correspond to the base pair locations in the GenBank sequence for the *Marinobacter aquaeolei* VT8 complete genome (GenBank: CP000514).

| **Time Point** | **Sample #** | **Start** | **End** | **Length** |
| --- | --- | --- | --- | --- |
| 0 hours (Fosmid) | 1 | 1,930,787 | 1,970,656 | 39.9 kb |
|  | 2 | 2,608,132 | 2,643,666 | 35.5 kb |
|  | 3 | 3,177,360 | 3,219,568 | 42.2 kb |
| 24 hours (Fosmid) | 1 | 2,535,901 | 2,567,306 | 31.4 kb |
|  | 2 | 962,181 | 1,001,405 | 39.2 kb |
|  | 3 | 1,881,833 | 1,915,757 | 33.9 kb |
| 48 hours (Fosmid) | 1 | 1,885,857 | 1,926,221 | 40.4 kb |
|  | 2 | 1,885,854 | 1,926,222 | 40.4 kb |
|  | 3 | 1,885,857 | 1,926,222 | 40.4 kb |
| 96 hours (Fosmid) | 1 | 1,885,855 | 1,926,241 | 40.4 kb |
|  | 2 | 1,885,857 | 1,926,235 | 40.4 kb |
|  | 3 | 1,885,856 | 1,926,237 | 40.4 kb |
| 96 hours (Plasmid) | 1 | 1,885,857 | 1,888,294 | 2.6 kb |
|  | 2 | 1,885,857 | 1,888,294 | 2.6 kb |
|  | 3 | 1,885,857 | 1,888,296 | 2.6 kb |
